# The miR‐200 family differentially regulates sensitivity to paclitaxel and carboplatin in human ovarian carcinoma OVCAR‐3 and MES‐OV cells

**DOI:** 10.1016/j.molonc.2015.04.015

**Published:** 2015-05-16

**Authors:** Anamaria Brozovic, George E. Duran, Yan C. Wang, E. Brian Francisco, Branimir I. Sikic

**Affiliations:** ^1^ Stanford University School of Medicine, Oncology Division, Stanford, CA 94305, USA

**Keywords:** Carboplatin, Drug resistance, Epithelial mesenchymal transition, miRNA‐200, Paclitaxel

## Abstract

We studied the role of miRNA‐200 family members in cellular sensitivity to paclitaxel and carboplatin, using two ovarian cancer cell lines, OVCAR‐3 and MES‐OV, and their paclitaxel resistant variants OVCAR‐3/TP and MES‐OV/TP. Both resistant variants display a strong epithelial‐mesenchymal transition (EMT) phenotype, with marked decreases in expression of miR‐200c and miR‐141 in OVCAR‐3/TP, and down‐regulation of all five members of the miR‐200 family in MES‐OV/TP. Lentiviral transfection of inhibitors of miR‐200c or miR‐141 in parental OVCAR‐3 triggered EMT and rendered the cells resistant to paclitaxel and carboplatin. Conversely, the infection of OVCAR‐3/TP cells with retroviral particles carrying the miR‐200ab429 and 200c141 clusters triggered a partial mesenchymal to epithelial transition (MET). This partial MET was not sufficient to re‐sensitize OVCAR‐3/TP cells to paclitaxel. However, the miR‐200c/miR‐141 cluster transfectants became 6–8x resistant to carboplatin, an unexpected result, whereas miR‐200a/miR‐200b/miR‐429 had no effect. Transfecting the OVCAR‐3/TP GFP cells with specific miRNA mimics confirmed these data. MiR‐200c and miR‐141 mimics conferred resistance to carboplatin in MES‐OV/TP cells, similar to OVCAR‐3/TP, but sensitized MES‐OV to paclitaxel. Several genes involved in balancing oxidative stress were altered in OVCAR‐3/TP 200c141 cells compared to controls. The miR‐200 family plays major, cell‐context dependent roles in regulating EMT and sensitivity to carboplatin and paclitaxel in OVCAR‐3 and MES‐OV cells.

## Highlights

- Paclitaxel resistant ovarian cancer cells manifest EMT and decreases in miRNA‐200s.
- Inhibition of miRNA‐200c and 141 produces resistance to paclitaxel and carboplatin.
- Re‐introducing miRNA‐200s reverts EMT and re‐sensitizes MES‐OV/TP to paclitaxel.
- miRNA‐200 family have differential effects on carboplatin resistance.

**Abbreviations**
ALDH1A3
: aldehyde dehydrogenase 1A3
AKR1C1 and AKRIC4: aldo-keto reductase family 1 and 4
CDH6
: cadherin 6
COL5A1
: collagen type V alpha 1
COL6A1
: collagen type VI alpha 1
EPCAM
: epithelial cell adhesion molecule
EMT: epithelial to mesenchymal transition
FACS: fluorescence-activated cell sorting
FBLN2
: fibulin 2
HGSOC: high grade serous ovarian cancer
MET: mesenchymal to epithelial transition
MTHFD2
: methylenetetrahydrofolate dehydrogenase
miRNA or miR: microRNA
Nrf2
: nuclear factor erythroid 2 [NF-E2]-related factor 2
RRM2
: ribonucleoside-diphosphate reductase subunit M2
RT-qPCR: real time quantitative polymerase chain reaction
SCD
: stearoyl-CoA desaturase-1
TUBB3: tubulin β3 isotype (also TUBBIII)
TXNDC12
: thioredoxin domain containing 12
VCAM 1
: vascular cell adhesion protein 1

## 1 Introduction

MicroRNAs (miRNAs) are known to play key roles in the regulation of differentiation, cell growth and cell death in normal and malignant tissues (Zamore and Haley, 2005; Iorio et al., 2007). Members of the miRNA‐200 family (miR‐200a, miR‐200b, miR‐200c, miR‐429 and miR‐141) inhibit epithelial to mesenchymal transition (EMT), by directly targeting the transcription factors ZEB1 and SNAI2, which negatively regulate the expression of E‐cadherin (*CDH1*) (Park et al., 2008; Korpal et al., 2008). Furthermore, miR‐200 family members regulate the expression of several mesenchymal markers including Fibronectin (*FN1*) (Cochrane et al., 2009) and Vimentin (*VIM*) (Rabik and Dolan, 2007; Mongroo and Rustgi, 2010; Thiery, 2002). During EMT the intracellular adhesion complexes are disrupted, apico‐basal polarity is lost and cells become mesenchymal, motile, invasive, and resistant to standard chemotherapeutics (Tsai and Yang, 2013), such as the combination of paclitaxel and carboplatin (Pennington et al., 2010).

EMT is associated with the development of drug resistance in ovarian cancer cell models (Kanakkanthara and Miller, 2013), and these resistant variants exhibit decreased miR‐200 family content, a mesenchymal phenotype, and in some cases markedly increased growth *in vivo* compared to parental cell lines (Moisan et al., 2014). We explored the role of EMT/MET in response of two ovarian cancer cell lines, OVCAR‐3 and MES‐OV, and their taxane resistant variants, OVCAR‐3/TP and MES‐OV/TP to paclitaxel and carboplatin. We hypothesized that forced induction of miR‐200 family members in the drug‐resistant variants with decreased level of miR‐200s would trigger MET and sensitize cells to drug treatment. The effects of transfection of miR‐200 lentiviruses and molecular mimics were determined on cell phenotype, drug sensitivity, and global gene expression in the paclitaxel‐resistant variants. We also studied the effect of inhibitors of these miRNAs on the phenotype of drug sensitive parental cells.

## 2 Materials and methods

### 2.1 Chemicals

Paclitaxel was obtained from the National Cancer Institute (Bethesda, MD), dissolved in ethanol and stored at −20 °C. Carboplatin was purchased from Sigma–Aldrich (St. Louis, MO), dissolved in water and stored at −20 °C. Sulforhodamine B was purchased from Sigma–Aldrich, dissolved in 1% acetic acid, used as 0.4% (w/v) solution, and kept at room temperature.

### 2.2 Cell lines

Two parental human serous ovarian carcinoma cell line were utilized: OVCAR‐3 (American Type Culture Collection, ATCC HTB‐161), and MES‐OV, developed by the Sikic Laboratory and submitted to the ATCC (Moisan et al., 2014). We developed the paclitaxel resistant variants, OVCAR‐3/TP and MES‐OV/TP, by step‐wise selection with paclitaxel and PSC‐833 (valspodar), an inhibitor of paclitaxel transport due to the inhibition of P‐glycoprotein function, in order to select for non‐transporter models of taxane resistance (Moisan et al., 2014). The resistant variants display an EMT phenotype and resistance to taxane drugs. The two pairs of parental and resistant variants were subsequently transfected with GFP‐luciferase for *in vivo* imaging, and are thus designated OVCAR‐3 GFP, MES‐OV GFP, OVCAR‐3/TP GFP and MES‐OV/TP‐GFP (Moisan et al., 2014). The cells were grown in McCoy's medium supplemented with 10% fetal bovine serum (Gibco BRL Life Technologies, Grand Island, NY) and cultured in a humidified atmosphere of 5% CO_2_ at 37 °C.

### 2.3 Establishment of stable miR‐200c and miR‐141 inhibitor expressing cell lines

OVCAR‐3 and OVCAR‐3/TP GFP cells (2 × 10^4^) were plated in 24‐well tissue culture plates. Following 24 h incubation, the cells were infected with lentiviral particles containing a scrambled control, hsa‐miR‐200c miRNA inhibitor or hsa‐miR‐141 miRNA inhibitor (GeneCopoeia, Rockville, MD) in the presence of hexadimethrine bromide (Polybrene; Abbott Laboratories Corp., Abbott Park, IL) and centrifuged for 90 min at 2000 rpm at 37 °C. After the centrifugation the cells were incubated at 37 °C for several days. The medium was changed once 24 h after transduction. The expression of mCherry in cells was checked 48 h after the infection under a fluorescence microscope. MiRNA‐inhibitor/mCherry positive cells (OVCAR‐3 GFP/mCherry empty, OVCAR‐3/TP GFP/mCherry empty, OVCAR‐3 GFP/mCherry 200c Inh and OVCAR‐3 GFP/mCherry 141 Inh) were sorted up to three times in order to obtain at least 98% mCherry positive cells in the cell population.

### 2.4 Retroviral generation and establishment of stable miR‐200 family members/mCherry expressing cell lines

The retroviral vectors pEQ‐Pam3, pLMP mCherry, pLMP mCherry miR‐200ab429 and pLMP mCherry miR‐200c141 were kindly provided by Prof. Dr. Goodall, Centre for Cancer Biology, Adelaide, Australia. Retroviral particles expressing mCherry were prepared by using 2 × 10^7^ 293T cells plated in 175 cm^2^ T‐flasks. Subconfluent cells 24 h after seeding were co‐transfected with 15 μg packaging pEQ‐Pam3 and 15 μg of miR‐cluster/mCherry expressing vectors in the presence of CaCl*2*, 2× HBS and 25 μL chloroquine (Sigma–Aldrich). The medium with transfection mix was changed after 6 h and fresh medium was added. The supernatant was harvested 24–48 h after the virus infection, filtered through a 0.45 μm polyethersulfone filter, and ultracentrifuged at 19,500 rpm at 4 °C for 2 h 20 min. The virus pellet was resuspended in plain IMDM medium (Invitrogen Life Technologies, Grand Island, NY) and frozen at −80 °C. 2 x 10^4^ OVCAR‐3 GFP and OVCAR‐3/TP GFP cells were plated in 24 well plates. The cells were infected 24 h later with retroviral particles in the presence of hexadimethrine bromide (Polybrene; Abbott Laboratories) and centrifuged at 2000 rpm at 37 °C for 90 min. After the centrifugation the cells were left in the incubator at 37 °C for several days. The medium was changed once 24 h after transduction. The expression of mCherry in successfully infected cells was checked 48 h after the infection with a fluorescence microscope. mCherry positive cells (OVCAR‐3 GFP/mCherry empty, OVCAR‐3/TP GFP/mCherry empty, OVCAR‐3/TP GFP/mCherry c141 and OVCAR‐3/TP GFP/mCherry ab429) were sorted up to three times in order to obtain at least 98% mCherry positive cells in the cell population.

### 2.5 Establishment of transient miR‐200 family members expressing cells by mimic transfection

DharmaFECT transfection reagent (Thermo Fisher Scientific, Waltham, MA) was combined with mirVana miRNA mimic 200a, 200b, 200c, 429, 141 or scrambled negative control (Ambion Life Technologies, Austin, TX) at a concentration of 50 nM, and incubated in OPTI‐MEM (GIBCO Life Technologies) for 20 min prior to addition to OVCAR‐3/TP GFP cells. Cells were incubated at 37 °C for 48 h, then seeded for cell survival assay and collected for RNA and FACS analysis.

### 2.6 Determination of cell survival

The sensitivity of cells to paclitaxel and carboplatin was determined using the SRB colorimetric assay (Skehan et al., 1990). Briefly, cells were seeded in 96‐well tissue culture plates (2.5 or 5 × 10^3^ cells/well) and allowed to attach overnight, followed by drug treatment with different concentrations of paclitaxel and carboplatin. Following 72–120 h drug incubations, the cells were fixed in 10% (w/v) trichloroacetic acid (TCA) overnight at 4 °C. The next day the cells were washed with distillated water, left to dry and then stained for 30 min with SRB reagent. Following three washes with 0.1% (w/v) acetic acid, the complex of SRB and cell proteins was dissolved with 10 mM Tris buffer, and absorbance was measured using a microplate reader at 570 nm (Molecular Devices, Sunnyvale, CA).

### 2.7 Western blot analysis

The cells were trypsinized and harvested by centrifugation, washed with PBS and resuspended in sonication buffer (20 mM Tris/HCl, pH 8.5, 1 mM EDTA, 5% glycerin, 1 mM DTT, 0.5 mM PMSF). After sonication (Vibra‐Cell, Sonic & Materials, Inc., Newtown, CT), cell debris was removed by centrifugation (15 min, 20,000 × *g* at 4 °C). The supernatants containing total cellular proteins were collected and protein concentration was determined using the Qubit Protein Assay kit read on the Qubit 2.0 Fluorometer (Invitrogen Life Technologies). 30 μg of total cellular proteins were loaded onto an Any kD^™^ Mini‐PROTEAN TGX Precast Gel and run for 30 min at 200 mA (Bio‐Rad, Hercules, CA). Separated proteins were transferred onto a 0.2 μm nitrocellulose membrane using the Trans‐Blot Turbo Transfer System (Bio‐Rad). Following a 1 h incubation in blocking buffer (5% nonfat dry milk, 0.1% Tween 20 in TBS), the membranes were exposed to the following primary antibodies: an anti‐TUBBIII (anti‐TUBB3) monoclonal (class III beta‐tubulin, clone TUJ1 (Covance, Princeton, NJ), anti‐pan‐alpha tubulin (clone DM1A, Sigma–Aldrich), an anti‐cleaved caspase‐3 polyclonal antibody (Cell Signaling Technology, Danvers, MA), and an anti‐PARP polyclonal (Roche, Indianapolis, IN) with incubation times optimized for each antibody. Antibodies were recognized by species‐specific secondary antibodies conjugated to horseradish‐peroxidase (Bio‐Rad), and proteins were visualized with Clarity Western ECL substrate (Bio‐Rad) according to the manufacturer's protocol. Equal protein loading was confirmed by re‐exposing the nitrocellulose membrane to an anti‐GADPH antibody (Santa Cruz Biotechnology, Santa Cruz, CA).

### 2.8 Flow cytometric analysis (FACS)

Cells were harvested using the non‐enzymatic dissociation buffer Versene (Life Technologies) and collected by centrifugation. Cells were exposed to an anti‐CD324 antibody (E‐cadherin, clone 180224, R & D Systems, Minneapolis, MN). For intracellular staining, cells were exposed to Flow Cytometry Fixation Buffer (R & D Systems) for 20 min at 4 °C in the dark, followed by the addition of Flow Cytometry Permeabilization buffer for 5 min at room temperature (R & D Systems). Cells were exposed to anti‐Vimentin (clone RV202) and anti‐Fibronectin antibodies (clone 10, BD Biosciences, San Jose, CA, used also for western blot) all at optimized antibody dilutions. Primary antibodies were recognized by PE‐conjugated goat anti‐mouse secondary antibodies (Life Technologies) on an LSR II Flow Cytometer (BD Biosciences).

### 2.9 Real‐time quantitative PCR (qPCR)

Total RNA was isolated from sub‐confluent growing cells with the use of Nucleo Spin miRNA Kit (Macherey‐Nagel, Bethlehem, PA), and 1 μg RNA was used for first‐strand cDNA synthesis by using the miScript II RNA Kit (Qiagen, Valencia, CA) according to the manufacturer's protocol. Real‐time PCR using SYBR Green dye was performed to detect mRNA expression (miScript SYBR Green PCR Kit, Qiagen). For detection of miR‐200 family members the miScript Primer Assay (Qiagen) was used according to manufacturer's instructions, with *RNU6* gene (Qiagen) used as an internal loading control. E‐cadherin (*CDH1*), Fibronectin (*FN1*), Vimentin (*VIM*), *ZEB1, ZEB2 and SNAI2* were amplified by 1× SYBR Universal PCR master mix (Applied Biosystems, Life Technologies) according to the manufacturer's instructions, and expression was normalized to the *GAPDH* gene. The primer sequences are listed in Table 1. All real‐time PCR reactions were run in QuantStudio 12K Flex Real‐Time PCR System (Life Technologies). Amplification efficiency was determined by serial dilutions. All reactions were performed in triplicate. Dissociation curves were recorded after each run to confirm primer specificity.

**Table 1:** Primer sequences for RT‐qPCR assays of gene expression.

Gene	Forward primer	Reverse primer
CDH1	TGAAGGTGACAGAGCCTCTGGAT	TGGGTGAATTCGGGCTTGTT
FN1	GGTGACACTTATGAGCGTCCTAAA	AACATGTAACCACCAGTCTCATGTG
SNAI2	ATGAGGAATCTGGCTGCTGT	CAGGAGAAAATGCCTTTGGA
TUBB3	CGAAGCCAGCAGTGTCTAAA	GCCTGGAGCTGCAATAAGAC
VIM	CCTTGAACGCAAAGTGGAATC	GACATGCTGTTCCTGAATCTGAG
ZEB1	AAGAAAGTGTTACAGATGCAGCTG	CCCTGGTAACACTGTCTGGTC
ZEB2	GCGGCATATGGTGACACACAA	CATTTGAACTTGCGATTACCTGC

### 2.10 Genomic profiling using the Illumina human HT‐12 expression BeadChip V4

Total RNA was isolated as described above, and 1 μg RNA was amplified and labeled with Illumina TotalPrep RNA amplification kit (Life Technologies, Ambion), and hybridized according to the manufacturer's protocol (Illumina GeneExpression Direct Hyb; illumina, San Diego, USA). Arrays were read by an Illumina Bead Chip Reader, and data were normalized by quantile normalization in GenePattern (http://genepattern.broadinstitute.org/gp/pages/index.jsf). The ratios of gene expression in OVCAR‐3/TP GFP/mCherry c141 versus OVCAR‐3/TP GFP/mCherry empty variants were determined, and the top 30 upregulated and downregulated genes were tabulated.

### 2.11 Statistical analysis

All data were analyzed by unpaired Student's *t*‐test, and expressed as the mean ± standard error of the mean. Data were considered significant when P values were lower than 0.05, and in the figures these are designated as * = *P* < 0.05, ** = *P* < 0.01 or *** = *P* < 0.001. Experiments were performed in triplicate and repeated at least twice.

## 3 Results

### 3.1 OVCAR‐3/TP cells demonstrate a mesenchymal phenotype associated with reduced miR‐200 content

**Figure 1:**
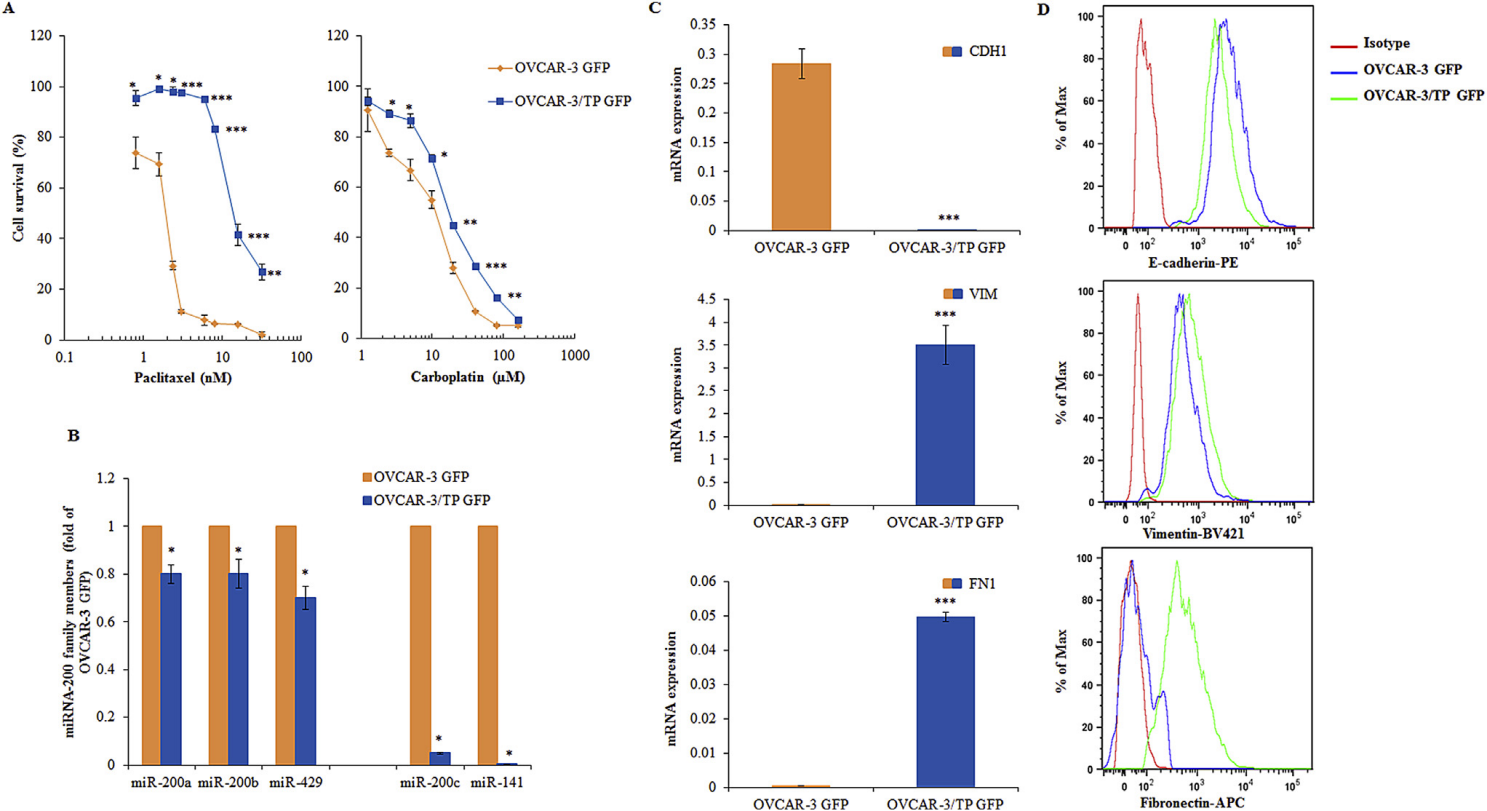
Characterization of OVCAR‐3 GFP and OVCAR‐3/TP GFP cell lines. (A) The cells were treated with different concentrations of paclitaxel (left panel) and carboplatin (right panel) during 72 h. Cells survival was measured by SRB assay. All data are expressed as the average percentage of survival values relative to an untreated control ± SD with significance determined between the indicated cell lines per paclitaxel or carboplatin concentration tested (*, P < 0.05; **, P < 0.01; ***, P < 0.001). (B) The constitutive expression of miR‐200 family members was determined by real time PCR 48 h after cell seeding, using the RNU6 gene as an internal loading control, and calculating the ratio of parental to resistant cells. (C) The constitutive expression of CDH1, FN1 and VIM was measured in cells by using real time PCR 48 h after seeding. All data are expressed as the average of at least three measurements. Significance was determined between the OVCAR‐3/TP GFP compared to the OVCAR‐3 GFP cell line (*, P < 0.05; **, P < 0.01; ***, P < 0.001), (B and C). (D) EMT marker proteins were measured in cells 48 h after seeding using flow cytometry, and representative histograms of 10,000 events per cell line for each channel (E‐cadherin‐PE, Vimentin‐Brilliant Violet 421, and Fibronectin‐APC) are shown. Results for OVCAR‐3 GFP cells are shown in orange and OVCAR‐3/TP‐GFP cells in blue.

The paclitaxel resistant OVCAR‐3/TP GFP cell line was previously described (Moisan et al., 2014) and is 8‐fold resistant to paclitaxel and 2‐fold cross‐resistant to carboplatin (Figure 1A). The paclitaxel resistance observed in the OVCAR‐3/TP variant is stable for more than 20 cell passages post‐drug selection with paclitaxel and PSC‐833 (data not shown). Down‐regulation of miR‐200 family members is associated with taxane resistance in this cell model (manuscript in preparation) and we confirmed these data in the GFP‐expressing cell lines. We observed significant down‐regulation of all members of the miR‐200 family in OVCAR‐3/TP GFP compared to OVCAR‐3 GFP cells, but miR‐200c and miR‐141 were much lower than miR‐200a, miR‐200b and miR‐429 under identical experimental conditions (Figure 1B). We also confirmed decreased epithelial marker E‐cadherin content and increased levels of mesenchymal markers, Fibronectin and Vimentin at both the transcriptional (Figure 1C) and translational (Figure 1D) levels in OVCAR‐3/TP GFP compared to parental OVCAR‐3 GFP cells.

### 3.2 Establishment of OVCAR‐3 GFP cell lines with expression of miR‐200c or miR‐141 inhibitors

**Figure 2:**
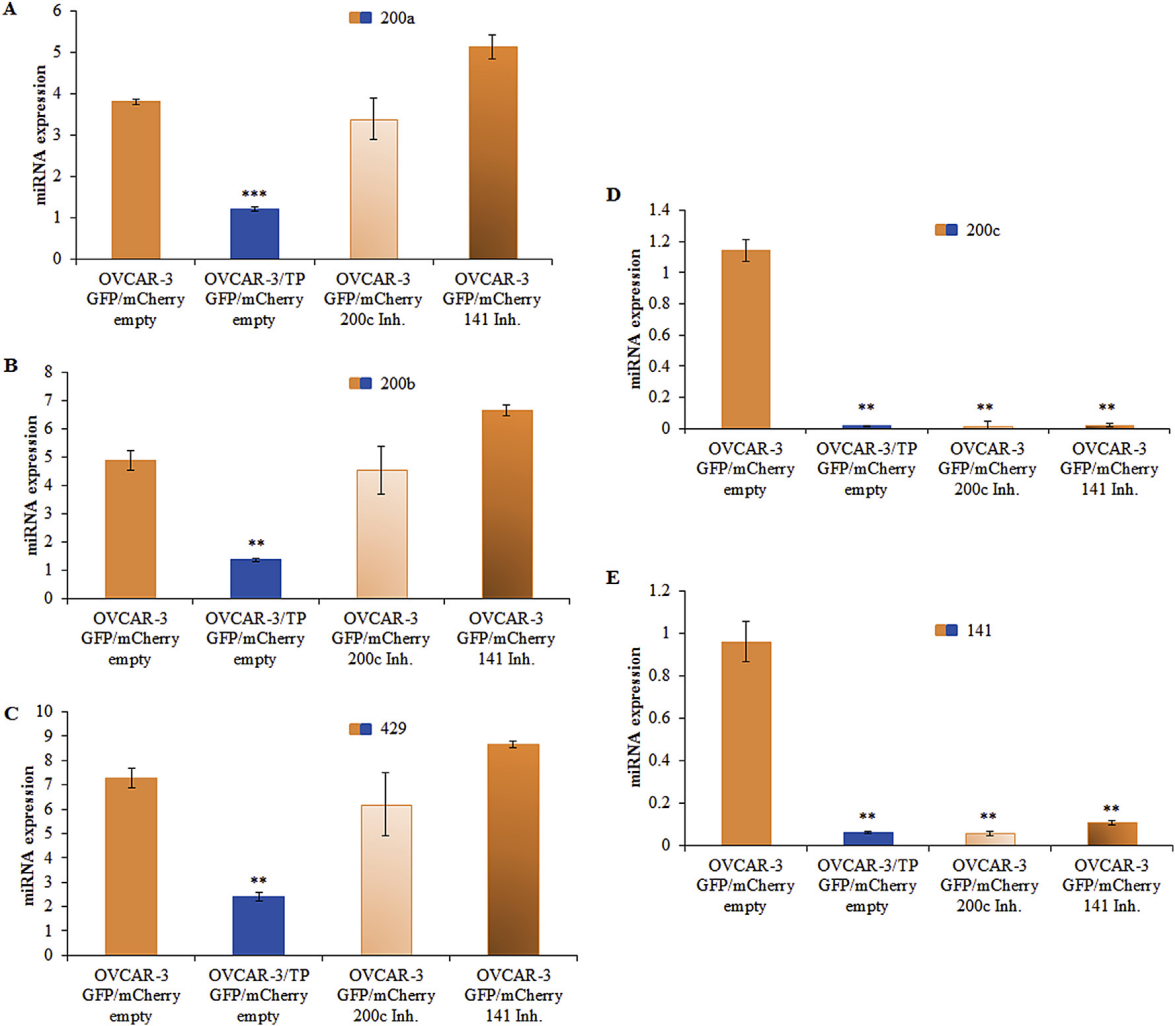
Generation of OVCAR‐3 GFP/mCherry cells with miR‐200c and miR‐141 inhibitors. Expression levels of miR‐200 family members were determined with real time PCR 48 h after cell seeding, relative to expression of the RNU6 gene as an internal loading control. (A) miR‐200a, (B) miR‐200b, (C) miR‐429, (D) miR‐200c, and (E) miR‐141. Representative data of three independent experiments are shown. All data are expressed as the average of at least three measurements. Significance was determined between the OVCAR‐3/TP GFP/mCherry empty, OVCAR‐3 GFP/mCherry 200c Inh. and OVCAR‐3 GFP/mCherry 141 Inh. compared to the OVCAR‐3 GFP/mCherry empty cell line (**, P < 0.01; ***, P < 0.001).

Since miR‐200c and miR‐141 are down‐regulated in the OVCAR‐3/TP GFP variant, we hypothesized that the down‐regulation of these miRNAs in OVCAR‐3 GFP cells could trigger cells to gain a mesenchymal phenotype and alter paclitaxel and carboplatin activity. We transduced OVCAR‐3 GFP cells with lentiviral particles carrying either empty, miR‐200c or miR‐141 miRNA inhibitor constructs. Additionally, we infected OVCAR‐3/TP GFP cells with lentiviral particles carrying an empty construct as a control. After cell sorting for GFP/mCherry positive cells by flow cytometry, we established OVCAR‐3 GFP/mCherry empty, OVCAR‐3/TP GFP/mCherry empty, OVCAR‐3 GFP/mCherry 200c Inh and OVCAR‐3 GFP/mCherry 141 Inh cell lines, and we measured the expression of miR‐200 family members by qPCR. As expected, the empty vector constructs did not affect miR‐200 expression in transduced OVCAR‐3 GFP and OVCAR‐3/TP GFP cells (1, 2), and the miR‐200c and miR‐141 inhibition was specific, with no effect observed in the expression levels of miR‐200a, miR‐200b and miR‐429 in OVCAR‐3 GFP/mCherry 200c Inh and OVCAR‐3 GFP/mCherry 141 Inh cells (Figure 2A–C).

There was almost complete inhibition of miR‐200c and miR‐141 in OVCAR‐3 GFP/mCherry 200c Inh and in OVCAR‐3 GFP/mCherry 141 Inh cells (Figure 2D, E). This reciprocal inhibition is probably due to the ZEB/miR‐200 double‐negative feedback loop (Burk et al., 2008; Brabletz and Brabletz, 2010) since the miRNA inhibitors block miRNA regulation of target gene expression. They are designed and optimized for miRNA loss of function. The inhibition of miR‐200c or miR‐141 in epithelial OVCAR‐3 GFP cell line resulted in two cell lines which express similar levels of miR‐200c and miR‐141 as the control mesenchymal OVCAR‐3/TP GFP/mCherry empty cell line.

### 3.3 Stable inhibition of miR‐200c or miR‐141 in epithelial OVCAR‐3 GFP cell line affects downstream targets

**Figure 3:**
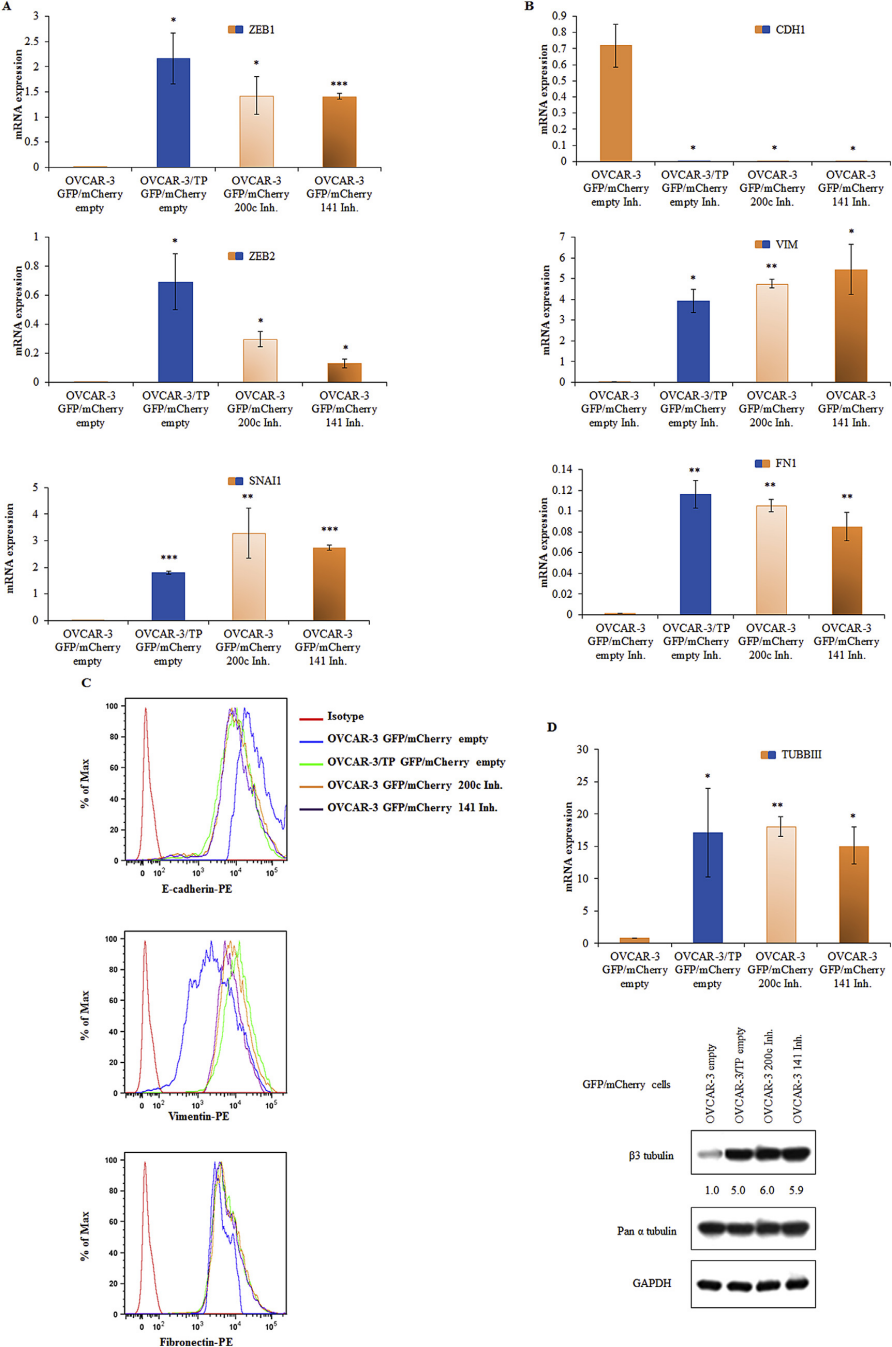
Expression of miR‐200c or miR‐141 inhibitors in OVCAR‐3 GFP/mCherry cells suppress the expression of EMT genes. (A) ZEB1, ZEB2 and SNAI2 were determined in cells by real time PCR 48 h after cell seeding. (B) CDH1, VIM and FN1 were determinate by real time PCR 48 h after seeding. Representative data of three independent experiments are presented. All data are expressed as the average of at least three measurements. Significance was determined between the OVCAR‐3/TP GFP/mCherry empty, OVCAR‐3 GFP/mCherry 200c Inh. and OVCAR‐3 GFP/mCherry 141 Inh. compared to the OVCAR‐3 GFP/mCherry empty cell line (*, P < 0.05; **, P < 0.01; ***, P < 0.001). (C) Flow cytometry was used to determine the expression of EMT marker proteins 48 h after cell seeding. Three independent experiments were performed and representative FACS histograms of 10,000 events are shown for each protein all recognized by a PE‐conjugated secondary antibody. (D) Expression levels of TUBBIII were determined with real time PCR 48 h after cell seeding relative to expression of the GAPDH gene as an internal loading control. Significance was determined between the OVCAR‐3/TP GFP/mCherry empty, OVCAR‐3 GFP/mCherry 200c Inh. and OVCAR‐3 GFP/mCherry 141 Inh. compared to the OVCAR‐3 GFP/mCherry empty cell line (*, P < 0.05; **, P < 0.01). The constitutive expression of TUBB3 was determined by western blot analysis. The samples were collected 48 h after the seeding. Pan α‐tubulin and GAPDH were used as loading controls. A representative blot of two independent experiments is shown.

It is known that the expression of *ZEB1*, *ZEB2* and *SNAI2* inversely correlates with the expression of miR‐200 family members (Burk et al., 2008; Korpal et al., 2008; Park et al., 2008). As expected, *ZEB1*, *ZEB2* and *SNAI2* are up‐regulated in OVCAR‐3 GFP/mCherry 200c Inh and OVCAR‐3 GFP/mCherry 141 Inh cell lines relative to OVCAR‐3 GFP/mCherry cells (Figure 3A). Furthermore, inhibition of miR‐200c and miR‐141 resulted in the down‐regulation of E‐cadherin and the up‐regulation of Vimentin and Fibronectin transcripts (Figure 3B) and proteins (Figure 3C) compared to OVCAR‐3 GFP/mCherry cells. These data provide further evidence of miR‐200c and miR‐141 regulation of EMT markers.

Prior studies have found that the miR‐200 family controls the expression of the class III β‐tubulin (TUBB3; TUBBIII) (Leskela et al.), (Prislei et al., 2013). We determined the expression of TUBB3 in these cell lines and found that the inhibition of miR‐200c and miR‐141 resulted in the up‐regulation of this β‐tubulin isotype (Figure 3D). The levels were 6‐fold higher than the empty vector controls, and 1.2‐fold higher than levels found in paclitaxel resistant OVCAR‐3/TP cells.

### 3.4 Induced EMT of OVCAR‐3 GFP cells resulted in resistance to paclitaxel

**Figure 4:**
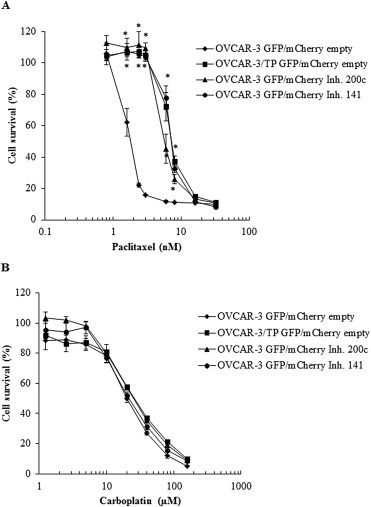
OVCAR‐3 GFP/mCherry cells with expression of miR‐200c and miR‐141 inhibitors are resistant to paclitaxel but not carboplatin under identical experimental conditions. (A) OVCAR‐3 GFP/mCherry empty Inh., OVCAR‐3/TP GFP/mCherry empty Inh., OVCAR‐3 GFP/mCherry 200c Inh. and OVCAR‐3 GFP/mCherry 141 Inh. cells were treated with different concentrations of paclitaxel. Cell survival was measured at 72 h by SRB. (B) Cells were treated with different concentrations of carboplatin. Cell survival was measured at 72 h by SRB. Representative data of three independent experiments are shown. All data are expressed as the average percentage of survival values relative to an untreated control ± SD with significance determined between the OVCAR‐3/TP GFP/mCherry empty, OVCAR‐3 GFP/mCherry 200c Inh. and OVCAR‐3 GFP/mCherry 141 Inh. compared to the OVCAR‐3 GFP/mCherry empty cell line per paclitaxel or carboplatin concentration tested (*, P < 0.05).

The inhibition of miR‐200c and miR‐141 in OVCAR‐3 GFP cells rendered these cells 4‐fold resistant to paclitaxel compared to OVCAR‐3 GFP empty vector controls (Figure 4A). Furthermore, the inhibition of miR‐200c and miR‐141 in OVCAR‐3 GFP cells resulted in decreased sensitivity to carboplatin although that effect is not apparent with 72 h exposure to carboplatin (Figure 4B). If the duration of the carboplatin exposure is increased up to 120 h, inhibition of miR‐200c and miR‐141 resulted in greater than 10‐fold resistance to carboplatin (Supplementary Figure 1). Thus, inhibition of miR‐200c and miR‐141 resulted in resistance to both paclitaxel and carboplatin in parental OVCAR‐3 GFP cells.

### 3.5 Introduction of miR‐200 family members in OVCAR‐3/TP GFP cells

**Figure 5:**
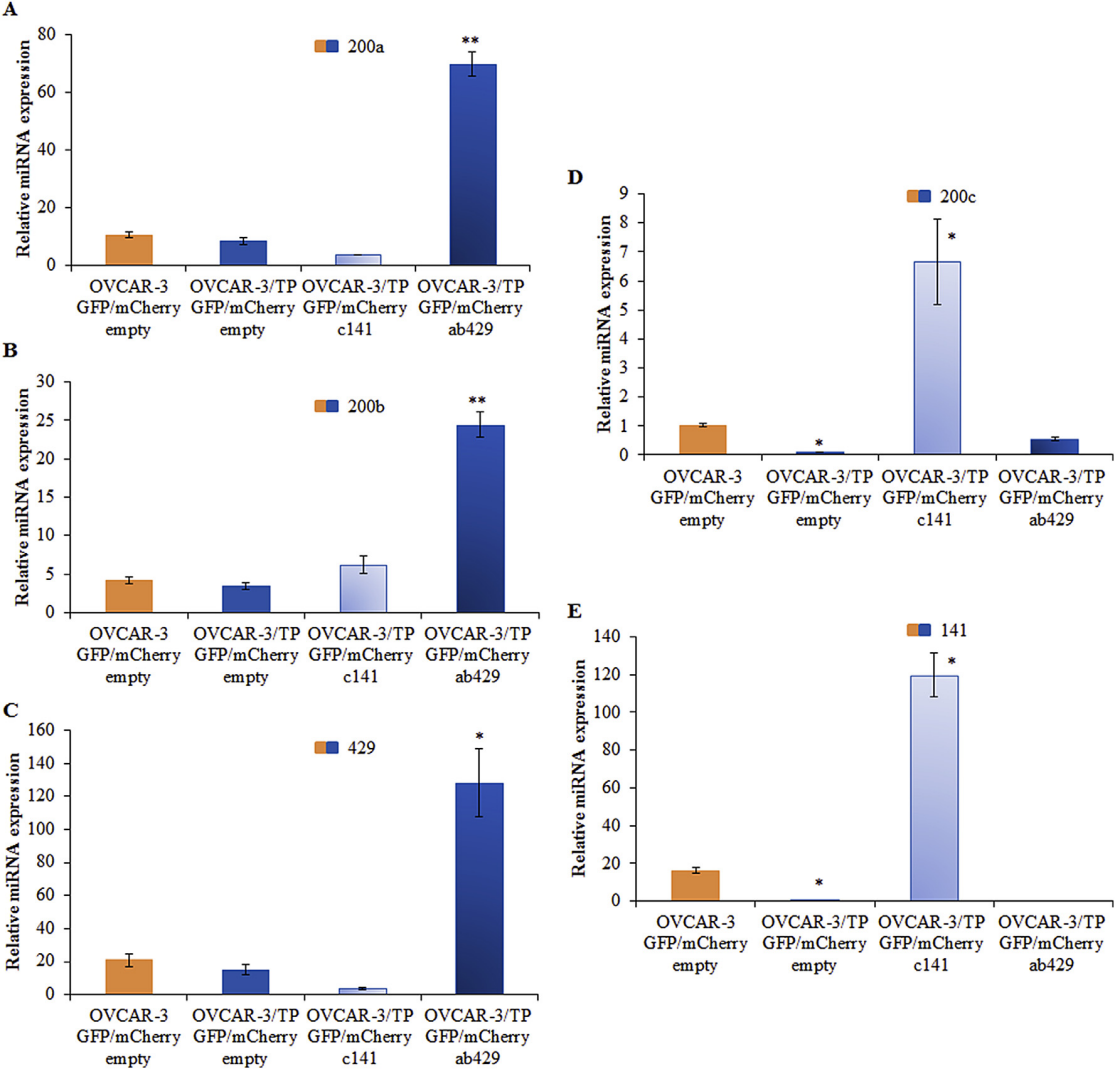
Generation of OVCAR‐3/TP GFP/mCherry cells with expression of ab429 and c141 cassettes. Total RNA was isolated from OVCAR‐3 GFP/mCherry empty, OVCAR‐3/TP GFP/mCherry empty, OVCAR‐3/TP GFP/mCherry ab429 and OVCAR‐3/TP GFP/mCherry c141 cells 48 h after cell seeding. The expression of (A) miR‐200a (200a), (B) miR‐200b (200b), (C) miR‐429 (429), (D) miR‐200c (200c), and (E) miR‐141 (141) was determined. Representative data of three independent experiments are shown. Significance was determined between the OVCAR‐3/TP GFP/mCherry empty compared to OVCAR‐3 GFP/mCherry empty, and OVCAR‐3/TP GFP/mCherry c141 and OVCAR‐3/TP GFP/mCherry ab429 compared to the OVCAR‐3/TP GFP/mCherry empty cell line (*, P < 0.05; **, P < 0.01).

MiR‐200 family members are generated from two distinct transcripts. MiR‐200a/miR‐200b/miR‐429 is derived from chromosome 1, and miR‐200c/miR‐141 is derived from chromosome 12 (Griffiths‐Jones et al., 2006). We studied the possible role of these two transcripts on the drug resistance of OVCAR‐3/TP GFP cells by transducing them with retroviral particles carrying either an empty mCherry‐retroviral construct, or the miR‐200ab429 (ab429) and 200c141 (c141) clusters. After sorting for GFP/mCherry positive cells, the transduced cells carrying the ab429 cluster showed significant increases in miR‐200a, miR‐200b and miR‐429 compared to controls, with no changes in expression of miR‐200c and miR‐141 (Figure 5A–C). Furthermore, retroviral‐infected cells carrying the c141 cluster displayed significant increases of miR‐200c (Figure 5D) and miR‐141 (Figure 5E) compared to controls, with no significant changes in expression of miR‐200a, miR‐200b and miR‐429 compared to OVCAR‐3/TP GFP/mCherry empty cells.

**Figure 6:**
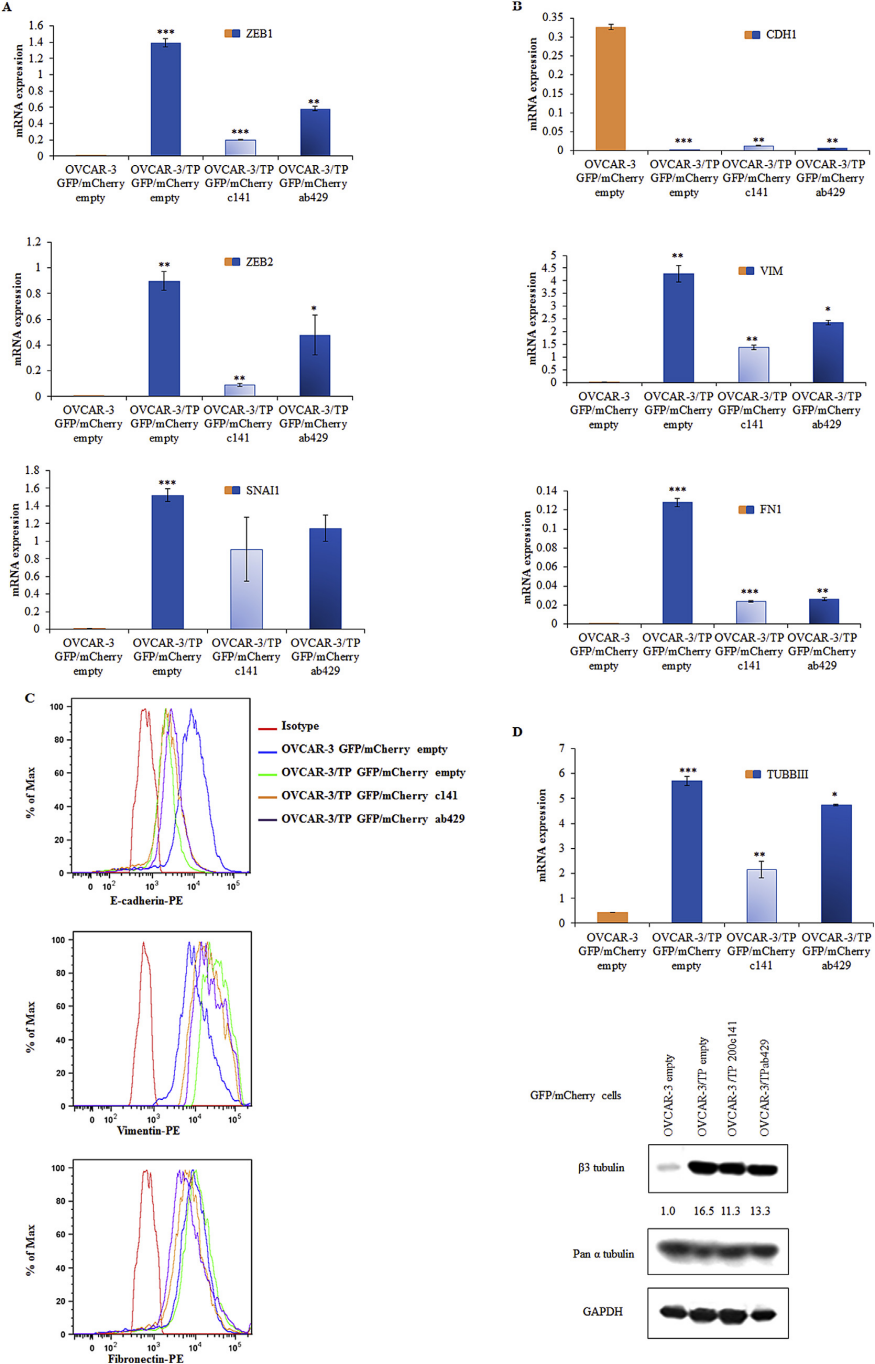
Up‐regulation of ab429 and c141 cassettes in OVCAR‐3/TP GFP/mCherry cells regulates the expression of EMT markers. (A) The expression of ZEB1, ZEB2 and SNAI2, and (B) CDH1, FN1 and VIM in growing cells was determined by RT‐qPCR. Representative data of three independent experiments are shown. Expression levels of indicated genes were determined with real time PCR 48 h after cell seeding relative to expression of the GAPDH gene as an internal loading control. Significance was determined between the OVCAR‐3/TP GFP/mCherry empty compared to OVCAR‐3 GFP/mCherry empty, and OVCAR‐3/TP GFP/mCherry c141 and OVCAR‐3/TP GFP/mCherry ab429 compared to the OVCAR‐3/TP GFP/mCherry empty cell line (*, P < 0.05; **, P < 0.01; ***, P < 0.001). (C) The expression of EMT proteins was determined by flow cytometry, and representative FACS histograms of 10,000 events are shown. Anti‐E‐cadherin and Fibronectin antibodies were recognized by a PE‐conjugate, and Vimentin expression was determined using a Brilliant Violet 421 (BV421)‐conjugated secondary antibody. (D) Expression levels of TUBBIII were determined with real time PCR 48 h after cell seeding relative to expression of the GAPDH gene as an internal loading control. Significance was determined between the OVCAR‐3/TP GFP/mCherry empty compared to OVCAR‐3 GFP/mCherry empty, and OVCAR‐3/TP GFP/mCherry c141 and OVCAR‐3/TP GFP/mCherry ab429 compared to the OVCAR‐3/TP GFP/mCherry empty cell line (*, P < 0.05; **, P < 0.01; ***, P < 0.001). The constitutive expression of TUBB3 was determined by western blot analysis. The samples were collected 48 h after the seeding. Pan α‐tubulin and GAPDH were used as loading controls. A representative blot of two independent experiments is shown.

The introduction of either the c141 or ab429 clusters in OVCAR‐3/TP GFP cells resulted in the down‐regulation of *ZEB1*, *ZEB2* and *SNAI2* compared to OVCAR‐3/TP GFP/mCherry empty controls (Figure 6A). However, these reductions did not attain the almost undetectable levels of *ZEB1*, *ZEB2* and *SNAI2* in the OVCAR‐3 GFP/mCherry empty cell line (Figure 6A). Similarly, expression of EMT markers was affected with the introduction of the c141 and ab429 clusters in OVCAR‐3/TP GFP (Figure 6B, C), but never achieving OVCAR‐3 GFP levels. Moreover, there was minimal decrease in TUBB3 levels with re‐introduction of miR‐200 family members to OVCAR‐3/TP cells (Figure 6D), further evidence that the miRNA‐triggered mesenchymal to epithelial transition (MET) in these cells is only partial.

### 3.6 Maintenance of increased expression of miR‐200 family members influences cellular responses to carboplatin but not to paclitaxel

**Figure 7:**
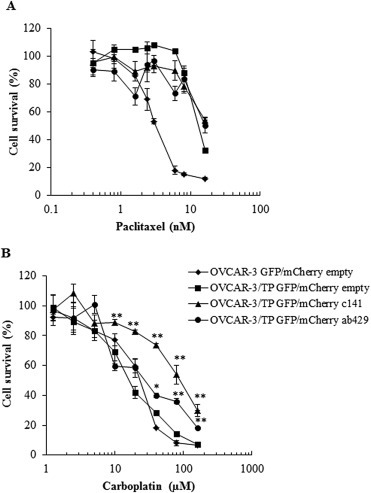
Up‐regulation of miR‐200c and miR‐141 increase survival of OVCAR‐3/TP GFP cells to carboplatin. (A) The cells were treated with different concentrations of paclitaxel or (B) carboplatin during 72 h. Cell survival was measured by SRB assays. Representative data of three independent experiments are shown. All data are expressed as the average percentage of survival values relative to an untreated control ± SD with significance determined between the indicated cell lines per paclitaxel or carboplatin concentration tested (*, P < 0.05; **, P < 0.01).

We hypothesized that the re‐introduction of miR‐200 family members in the OVCAR‐3/TP GFP cell line would re‐sensitize these cells to paclitaxel. However, there was no change in paclitaxel resistance after transduction of c141 or ab429 clusters in OVCAR‐3/TP GFP/mCherry c141 and OVCAR‐3/TP GFP/mCherry ab429 (Figure 7A). Unexpectedly, we observed 4‐fold resistance to carboplatin in the c141 but not ab429 cells (Figure 7B).

**Figure 8:**
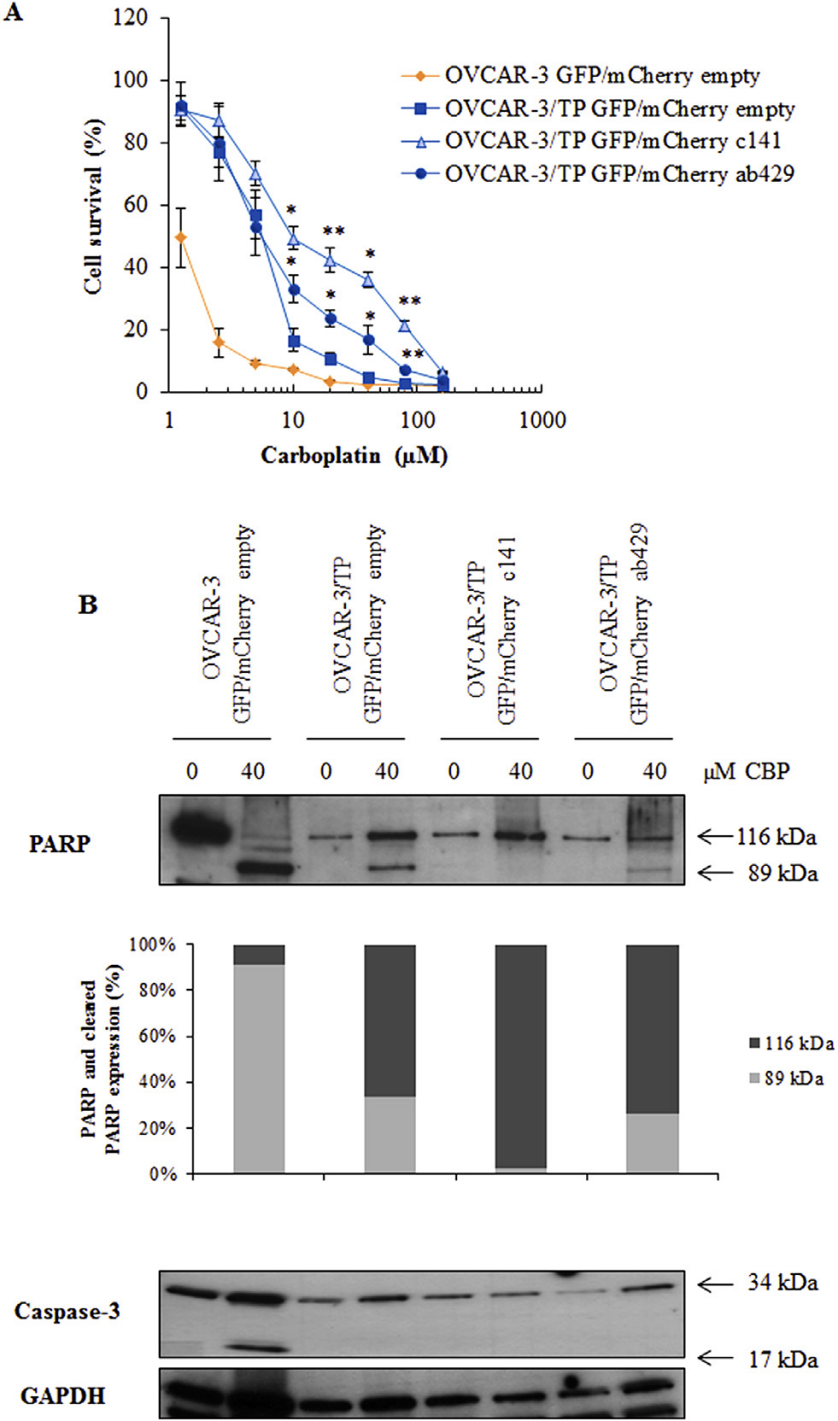
Up‐regulation of miR‐200c and miR‐141 in OVCAR‐3/TP GFP cells increase survival of cells upon long carboplatin treatment. (A) The cells were treated with different concentrations of carboplatin during 120 h. Cell survival was measured by SRB assays. All data are expressed as the average percentage of survival values relative to an untreated control ± SD with significance determined between the indicated cell lines per carboplatin concentration tested (*, P < 0.05; **, P < 0.01). (B) Cells were harvested 120 h after the treatment with the indicated concentrations of carboplatin. Non‐cleaved (116 kDa) and cleaved (89 kDa) PARP were determined by western blot. Densitometric analysis data are expressed as a ratio between PARP fragments and GAPDH that was used as an internal loading control. Non‐cleaved (34 kDa) and cleaved (17 kDa) caspase‐3 were determined using specific antibodies. Data from one of two experiments that yielded similar results are presented.

We confirmed the induction of carboplatin resistance by miR‐200c and miR‐141 in OVCAR‐3/TP cells using 120 h carboplatin exposure (Figure 8A). We then performed western blot analysis of specific cell death markers, PARP and caspase‐3 cleavage, following treatment with 40 μM of carboplatin for 120 h. PARP cleavage was detected in all cells except OVCAR‐3/TP GFP/mCherry c141, consistent with the cell survival data (Figure 8B). OVCAR‐3 GFP/mCherry empty controls are highly sensitive to carboplatin treatment and PARP protein is cleaved completely. Cleavage of PARP was detected in the OVCAR‐3/TP GFP/mCherry empty controls, but was much less compared to OVCAR‐3 GFP/mCherry empty cells, and slightly greater than OVCAR‐3/TP GFP/mCherry ab429 cells (densitometry, Figure 8B). Cleavage of caspase‐3 was found only in the most drug‐sensitive OVCAR‐3 GFP/mCherry empty cell line (Figure 8B).

**Figure 9:**
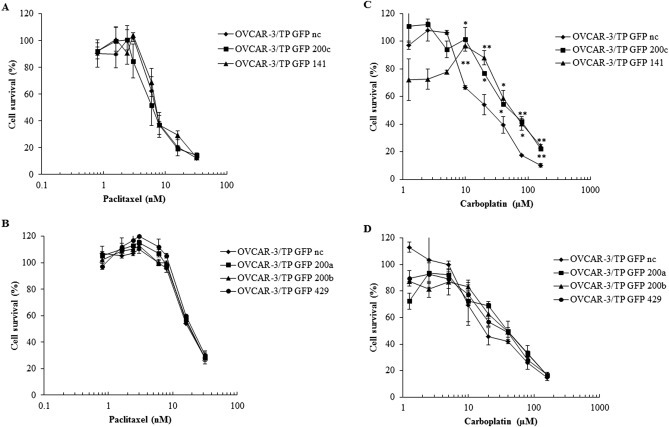
Transient transfection of OVCAR‐3/TP GFP cells with miR‐200 family member mimics confirmed cell survival data obtained with overexpression of miR‐200 family members upon treatment with paclitaxel or carboplatin. Cells were seeded for survival assays 48 h after transfection with mimics of (A, C) miR‐200c or miR‐141, (B, D) miR‐200a, miR‐200b or mR‐429. They were treated with different concentrations of paclitaxel (A, B) or carboplatin (C, D) 24 h later. Cell survival was measured 72 h later by SRB assays. Representative data of three independent experiments are shown. All data are expressed as the average percentage of survival values relative to an untreated control ± SD with significance determined between the negative control (nc) and cell transfected with indicated mimic per paclitaxel or carboplatin concentration tested (*, P < 0.05; **, P < 0.01).

We introduced specific miR‐200 mimics by transient transfection in OVCAR‐3/TP GFP cells. The addition of miR‐200a, miR‐200b, miR‐429, miRN200c or miR‐141 did not change their response to paclitaxel (Figure 9A, B). However, when the OVCAR‐3/TP GFP cells were transfected with miR‐200c or miR‐141 mimics, they became 2.3‐fold resistant to carboplatin compared to negative controls (Figure 9C). In contrast, transfection with miR‐200a, miR‐200b or miR‐429 mimics had a minimal effect on carboplatin sensitivity (Figure 9D).

FACS analysis of EMT markers upon transfection of OVCAR‐3/TP GFP cells with miR‐200c and miR‐141 mimics demonstrated up‐regulation of E‐cadherin (Supplementary Figure 2A) and down‐regulation of Fibronectin and Vimentin (Supplementary Figure 2B, C). Similar effects were seen with transfections of miR‐200a, miR‐200b and miR‐429 mimics (Supplementary Figure 2A–C).

### 3.7 Increased expression of miR‐200c and miR‐141 in the paclitaxel resistant ovarian cancer MES‐OV/TP GFP cell variant affects cellular responses to carboplatin and paclitaxel

**Figure 10:**
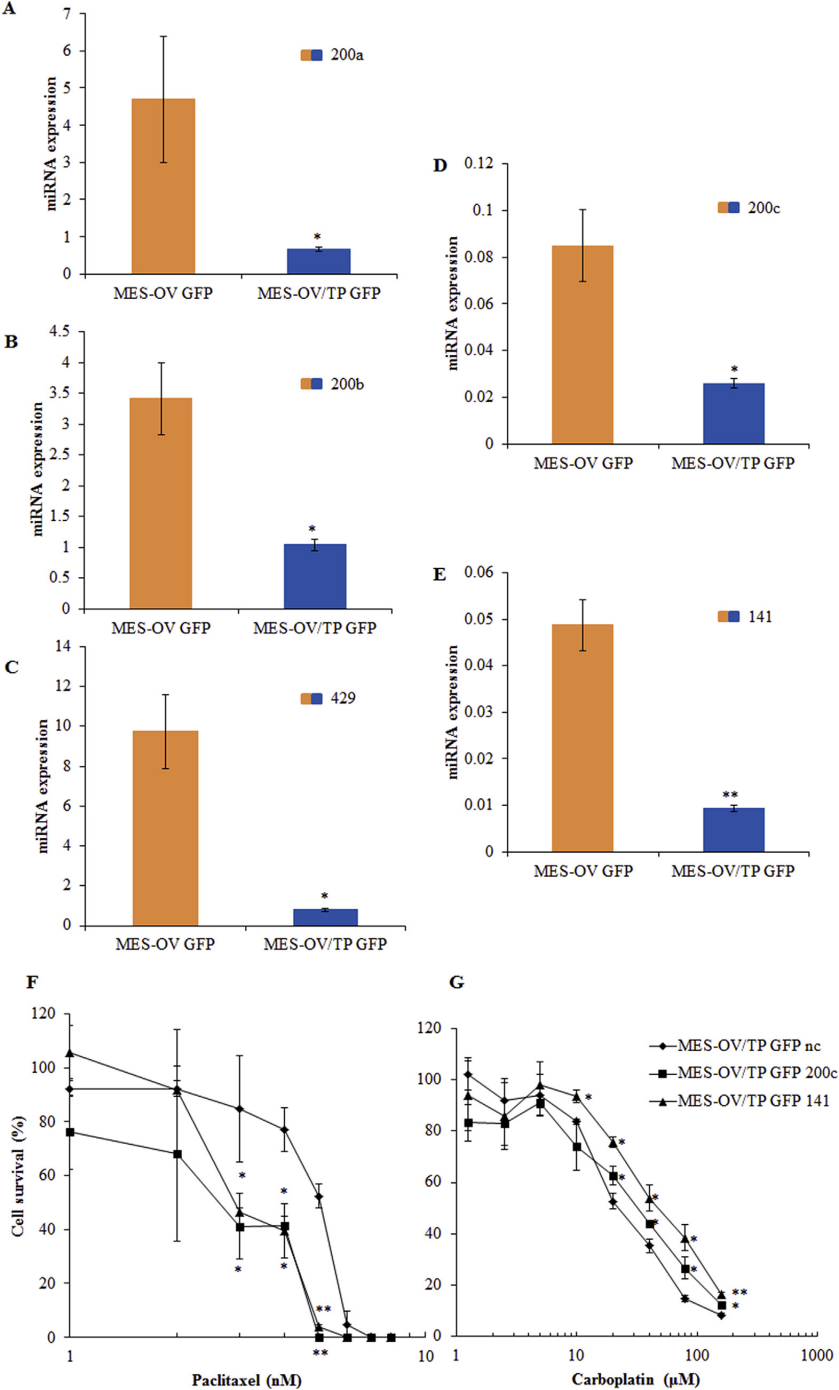
MES‐OV/TP cells are characterized with decreased expression of all miR‐200 family members. Transient transfection of MESOV/TP GFP cells with miR‐200c or miR‐141 mimics sensitize of cells to carboplatin. (A–E) The constitutive expression of miR‐200 family members, 200a (A), 200b (B), 429 (C), 200c (D) and 141 (E) was determined in cells by RT‐qPCR 48 h after seeding. (F, G) MES‐OV/TP GFP cells were seeded for cell survival assays 48 h after the transfection with miR‐200c or miR‐141 mimics. The cells were treated 24 h later with different concentrations of paclitaxel (F) or carboplatin (G), and cell survival was measured 72 h later by SRB. Representative data of three independent experiments are shown. All data are expressed as the average percentage of survival values relative to an untreated control ± SD with significance determined between the negative control (nc) and cell transfected with indicated mimic per paclitaxel or carboplatin concentration tested (*, P < 0.05; **, P < 0.01).

We studied the effects of transfected miR‐200c and miR‐141 mimics in another paclitaxel resistant variant, MES‐OV/TP GFP (Moisan et al., 2014). These cells have reduced miR‐200s (Figure 10A–E) and EMT (data not shown). MES‐OV/TP GFP cells transfected with miR‐200c or miR‐141 mimics became resistant to carboplatin (Figure 10G), similar to our findings in OVCAR‐3/TP cells. However, in contrast to OVCAR‐3/TP, re‐expression of miR‐200c and miR‐141 sensitized MES‐OV/TP cells to paclitaxel (Figure 10F). The miR‐141 mimic increased E‐cadherin in MES‐OV/TP cells, and both miR‐141 and miR‐200c mimics decreased Vimentin expression, indicating the induction of a partial MET (Supplementary Figure 3).

### 3.8 Genomic profiling of OVCAR‐3/TP versus OVCAR‐3/TP/c141 cells

We compared the microarray expression profiles of OVCAR‐3/TP GFP/mCherry empty and OVCAR‐3/TP GFP/mCherry c141 cells in order to identify genes that are differentially expressed in the transfected cells with resistance to carboplatin (Table 2). The MET induced by miR‐200bc/141 was confirmed by up‐regulation of *EPCAM* (epithelial cell adhesion molecule) and down‐regulation of *COL6A1* (collagen type VI alpha 1), *COL5A1* (collagen type V alpha 1), *CDH6* (cadherin 6), *FBLN2* (fibulin 2), and *VCAM1* (vascular cell adhesion molecule 1). Several redox genes are also altered, including up‐regulated *ALDH1A3* (an aldehyde dehydrogenase), *TXNDC12* (thioredoxin domain containing 12), *RRM2* (ribonucleotide reductase subunit 2) and *MTHFD2* (methylene tetrahydrofolate reductase 2), and down‐regulated *AKR1C1* and *AKRIC4* (aldo‐keto reductases), and *SCD* (stearoyl–CoA desaturase). These alterations implicate oxidative stress response genes in resistance to carboplatin in OVCAR‐3/TP GFP/c141 cells.

**Table 2:** Top 30 upregulated and 30 downregulated genes in OVCAR‐3/TP GFP/mCherry c141 variant compared to control OVCAR‐3/TP GFP/mCherry empty cells. Data are presented as ratios between OVCAR‐3/TP GFP/mCherry c141 and OVCAR‐3/TP GFP/mCherry empty variants.

OVCAR‐3/TP GFP/mCherry c141 top 30 upregulated	Ratio	Ratio	OVCAR‐3/TP GFP/mCherry c141 top 30 downregulated
ALDH1A3	4.79	−2.25	MXD4
EPCAM	3.39	−2.25	GPR162
TACSTD1	3.22	−2.26	OLFML2A
DMKN	3.01	−2.26	RARRES3
LOC100132240	2.99	−2.28	CAPN5
IL1R2	2.92	−2.29	MAMDC2
CDS1	2.81	−2.30	FOS
MAL2	2.80	−2.32	IFITM2
PKP2	2.69	−2.33	NNMT
CRISPLD2	2.67	−2.39	HBA2
FLG	2.65	−2.40	TMEM90B
SLC3A2	2.45	−2.40	TXNIP
C13ORF15	2.42	−2.42	CDH6
ZBED2	2.41	−2.46	LRIG1
KRT80	2.37	−2.49	VCAM1
S100A2	2.34	−2.53	MTSS1
OVOL2	2.23	−2.53	PLCH2
SLC7A5	2.23	−2.56	IGFBP4
ARL14	2.22	−2.61	FBLN2
NMU	2.17	−2.70	COL6A1
TGM2	2.16	−2.76	COL5A1
LOC647993	2.16	−2.78	ANXA4
LOC647987	2.13	−2.79	PLAT
SERPINA1	2.12	−2.80	VASN
SERPINB7	2.08	−2.92	AKR1C4
RAC1	2.05	−2.93	C1S
C20ORF75	1.99	−3.17	SCD
CST6	1.96	−3.25	ADAMTS9
TGFA	1.96	−3.68	AKR1C3
TRIB3	1.95	−4.09	OLFML3

## 4 Discussion

Late diagnosis, the development of drug resistance, and the metastatic spread of ovarian cancer cells are responsible for the high frequency of ovarian cancer deaths, particularly the common high grade serous ovarian cancers (HGSOC) (Jemal et al., 2011). The miR‐200 family regulates EMT (Castilla et al., 2011) which plays a central role in cancer cell invasion and metastasis (Thiery, 2002; De Craene and Berx, 2013) and in drug resistance of many types of cancers (Cochrane et al., 2009; Li et al., 2009; Ceppi et al., 2010; Mateescu et al., 2011; Cittelly et al., 2012; Fu et al., 2013; Prislei et al., 2013).

We have previously developed taxane‐resistant variants, OVCAR‐3/TP and MES‐OV/TP, from the ovarian cancer cell lines OVCAR‐3 and MES‐OV, and shown that these variants manifest EMT and decreased miR‐200 family expression (Moisan et al., 2014; unpublished data; and Figure 1). We therefore studied the effects of up‐ and down‐regulation of miR‐200 family members in our models on cellular sensitivity to paclitaxel and carboplatin, the two major drugs used in the chemotherapy of HGSOC. Transduction of parental OVCAR‐3/GFP cells with lentiviral particles carrying either a miR‐200c or miR‐141 inhibitor produced a partial mesenchymal transition (EMT), up‐regulation of the class III β‐tubulin isotype, and 4–5‐fold resistance to paclitaxel (2, 3, 4). Thus, inhibition of the miR‐200 family in drug sensitive parental cells recapitulated the phenotype of our taxane‐selected taxane resistant cells. High levels of TUBB3 have previously been reported to be associated with taxane resistance (Kavallaris et al., 1997; Burkhart et al., 2001). Leskela et al. (2010) found that tumors with high levels of TUBB3 protein have significantly decreased miR‐200 expression, and the strongest association was with decreased miR‐141, miR‐429 and miR‐200c (Leskela et al.), (Prislei et al., 2013).

Although down‐regulation of TUBB3 was reported to restore sensitivity to taxanes (Cochrane et al., 2009), as did up‐regulation of miR‐200a and miR‐141 (Mateescu et al., 2011), we found divergent results in our two taxane resistant variants which express high TUBB3 levels, OVCAR‐3/TP and MES‐OV/TP. We up‐regulated miR‐200 family members either with stable retroviral constructions containing miRNAs clusters or mimics by transient transfection. Despite achieving very high expression of each member of the miR‐200 family in OVCAR‐3/TP cells, we were unable to completely restore the epithelial phenotype to parental cell levels, decrease TUBB3 expression, and increase sensitivity to paclitaxel. Thus, restoration of miR‐200 in the resistant cells was not sufficient to reverse the established mesenchymal phenotype and paclitaxel resistance in these cells (5, 6, 7). In contrast to OVCAR‐3/TP cells, the up‐regulation of miR‐200 in the paclitaxel‐selected ovarian MES‐OV/TP variant did result in substantial sensitization to paclitaxel (Figure 10), although MET was partial. Thus, the ability of the miR‐200 family to sensitize resistant cells to taxanes is cell context dependent. The OVCAR‐3/TP and MES‐OV/TP variants differ with regard to their parental cells and spectrum of EMT marker expression, as well as in their alterations of miR‐200 family members. Notably, MES‐OV/TP cells have a strong decrease of expression of all members of miR‐200 family, where the decrease is specifically for miR‐200c and miR‐141 in OVCAR‐3/TP cells (1, 10A–E). Moreover, step‐wise selection of resistant variants is likely to yield variants with more than one concurrent mechanism of resistance (unpublished observations).

Our observations with regard to resistance to carboplatin in relation to miR‐200s and resistance to taxanes have important implications for the treatment of cancers with these drugs. Taxane‐selected OVCAR‐3/TP cells are slightly cross‐resistant to carboplatin, but re‐expression of the miR‐200 family and miR‐200 mimics, particularly miR‐200c and miR‐141, further increased resistance to carboplatin, as evident from both cell survival and apoptotic endpoints (7, 8, 9C). Furthermore, it seems that the 200ab429 and 200c141 clusters appear to have different functions in cellular responses to carboplatin in OVCAR‐3/TP cells. This differential effect of the miRNA‐200 family clusters on chromosome 1 versus chromosome 12 is of interest and should be further investigated. Taxane‐selected MES‐OV/TP cells are not cross‐resistant to carboplatin. However, miR‐200c and miR‐141 mimics produced resistance to carboplatin in MES‐OV/TP, similar to their effects in OVCAR‐3/TP (Figure 10G). Thus, we show that miR‐200c and miR‐141 can increase resistance to carboplatin when introduced into both of these taxane‐resistant variants. The observation that miR‐200c and miR‐141 produce resistance to carboplatin while sensitizing MES‐OV/TP cells to paclitaxel is a strong indicator that the effects of these miRNAs on drug sensitivity are cell context dependent. In the case of our two ovarian cell models, these miR‐200s can have opposite effects on the two major cytotoxic drugs used in HGSOC. These results are consistent with those of van Jaarsveld et al. who showed that over‐expression of miR‐141 enhanced resistance to cisplatin in ovarian cancer A2780 cells (van Jaarsveld et al., 2013). Others reported an opposite effect, that over‐expression of miR‐200c in A2780 cells sensitized cells to cisplatin (Prislei et al., 2013). These authors suggested that the A2780 and OVCAR‐3 cell lines represent two different models of miR‐200c action, based upon the nuclear/cytoplasmic ratio of the transcriptional regulator HuR (Prislei et al., 2013). However, the OVCAR‐3 cells utilized by Prislei et al. (2013) have high expression of TUBB3 and relative resistance to paclitaxel (IC_50_ 22 nM), which differs from our parental OVCAR‐3 cells with low TUBB3 expression and high sensitivity (IC_50_ 2 nM) to paclitaxel (1, 3D).

We performed whole genome expression assays to explore differences between OVCAR‐3/TP/c141 cells with high miR‐200c and miR‐141 expression (and induced resistance to carboplatin) versus the control OVCAR‐3/TP cells with low miR‐200c and miR‐141 (Table 2). In addition to confirmed MET‐related gene expression, this analysis revealed alterations in several genes involved in balancing oxidative stress. Genes upregulated by the miR‐200c/141 construct included *ALDH1A3*, *TXNDC12*, *RRM2* and *MTHFD2*, while *AKR1C1* and *AKRIC4* and *SCD* were down‐regulated. Several of these genes have been implicated in cellular sensitivity to platinum drugs. Down‐regulation of *ALDH1A1* expression has sensitized chemoresistant tumors to cisplatin *in vitro* and in mouse models (Landen et al., 2010). *ALDH* was implicated in conveying tolerance to cisplatin in three malignant pleural mesothelioma cell lines (Cortes‐Dericks et al., 2014). Knockdown of *RRM2* significantly reversed cisplatin resistance in ovarian SKOV‐3/DDP cells (Zhang et al., 2013). *MTHFD2* was identified as a transcriptional target of Nrf2, that increases the expression of several cytoprotective genes (Jaramillo and Zhang, 2013). Overexpression of the three AKR1C isoforms was reported in cisplatin‐resistant phenotypes of ovarian cancer cells (Deng et al., 2002; Chen et al., 2005; Chen et al., 2008). This is in contrast to our reduced levels of *AKR1C1* and *AKRIC4* in OVCAR‐3/TP cells with up‐regulated miR‐200c/141. Among other genes that were down‐regulated by miR‐200c/141, *SCD* is described as an important player in tumorigenesis. Our data support the importance of the regulation of reactive oxygen species in cellular responses to platinum drugs (Brozovic et al., 2008; Brozovic et al., 2013) and the involvement of miR‐200 family members in regulating oxidative stress (Wiemer, 2011).

In summary, we confirm that the miR‐200 family has major roles in EMT and taxane resistance in taxane selected ovarian cancer cell variants, and that re‐introduction of miR‐200s was not sufficient to fully reverse the mesenchymal phenotype in these variants. Although miR‐200s were able to restore paclitaxel sensitivity in one of the variants, they did not do so in the other, and produced resistance to carboplatin in both. The divergent effects of miR‐200s on taxane and carboplatin cytotoxicity should be further investigated in ovarian cancers.

## Author disclosure information

The authors state no conflict of interest.

## Financial support

This study was supported by NIH Grant R01 CA 184968, the Croatian Visiting Scientist Fund, and the Brigitte Decre Ovarian Cancer Research Fund (all in support of research in the Sikic Laboratory at Stanford University).

## Supporting information



The following are the supplementary data related to this article:

Supplemental Figure 1 OVCAR‐3 GFP/mCherry cells with expression of miR‐200c and miR‐141 inhibitors are resistant to carboplatin. OVCAR‐3 GFP/mCherry empty, OVCAR‐3/TP GFP/mCherry empty, OVCAR‐3 GFP/mCherry 200c Inh. and OVCAR‐3 GFP/mCherry 141 Inh. Cells were seeded for survival assays, and 24 h later the cells were treated with different concentrations of carboplatin. Cell survival was measured 120 h later by SRB. Representative data of 3 independent experiments are shown. All data are expressed as the average percentage of survival values relative to an untreated control ± SD with significance determined between the OVCAR‐3/TP GFP/mCherry empty, OVCAR‐3 GFP/mCherry 200c Inh. and OVCAR‐3 GFP/mCherry 141 Inh. compared to the OVCAR‐3 GFP/mCherry empty per carboplatin concentration tested (*, P < 0.05).

Supplemental Figure 2 Transient transfection of OVCAR‐3/TP GFP with miR‐200a, miR‐200b, miR‐429, miR‐200c or miR‐141 mimics regulates protein expression of EMT markers. OVCAR‐3/TP GFP cells were transfected with negative control (nc), miR‐200a, miR‐200b, miR‐200c, miR‐429, or miR‐141 mimics. The EMT markers E‐cadherin (A), Vimentin (B), and Fibronectin (C) proteins were measured 24 h later by flow cytometry. The representative FACS histograms of 10,000 events per condition are shown in each panel. For easier interpretation of FACS histograms the results are presented as bar graphs (right panel) where y‐ax presents the fluorescence intensity.

Supplemental Figure 3 Transient transfection of MES‐OV/TP GFP cells with miR‐200c or miR‐141 mimics regulates EMT markers. MES‐OV/TP GFP cells were transiently transfected with miR‐200c or miR‐141 mimics. Cells were collected 48 h after the transfection, and expression of E‐cadherin (A), Vimentin (B), and Fibronectin (C) proteins was measured by flow cytometry. The representative FACS histograms of 10,000 events per condition are shown in each panel. For easier interpretation of FACS histograms the results are presented as bar graphs (right panel) where y‐ax presents the fluorescence intensity.

## Supplementary data 1

### 1.1

Supplementary data related to this article can be found at http://dx.doi.org/10.1016/j.molonc.2015.04.015.
