# Supplementary figures and images for: The miR‐200 family differentially regulates sensitivity to paclitaxel and carboplatin in human ovarian carcinoma OVCAR‐3 and MES‐OV cells
Source: Mol Oncol. 2015 May 16;9(8):1678–93. doi: 10.1016/j.molonc.2015.04.015 (PMC4788969; doi:10.1016/j.molonc.2015.04.015)

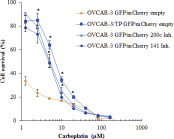

Supplement: Supplementary file 1 — Supplemental Figure 1 OVCAR‐3 GFP/mCherry cells with expression of miR‐200c and miR‐141 inhibitors are resistant to carboplatin. OVCAR‐3 GFP/mCherry empty, OVCAR‐3/TP GFP/mCherry empty, OVCAR‐3 GFP/mCherry 200c Inh. and OVCAR‐3 GFP/mCherry 141 Inh. Cells were seeded for survival assays, and 24 h later the cells were treated with different concentrations of carboplatin. Cell survival was measured 120 h later by SRB. Representative data of 3 independent experiments are shown. All data are expressed as the average percentage of survival values relative to an untreated control ± SD with significance determined between the OVCAR‐3/TP GFP/mCherry empty, OVCAR‐3 GFP/mCherry 200c Inh. and OVCAR‐3 GFP/mCherry 141 Inh. compared to the OVCAR‐3 GFP/mCherry empty per carboplatin concentration tested (*, P < 0.05). [file MOL2-9-1678-s001.jpg]

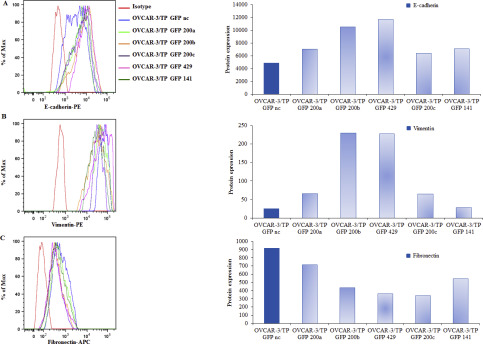

Supplement: Supplementary file 2 — Supplemental Figure 2 Transient transfection of OVCAR‐3/TP GFP with miR‐200a, miR‐200b, miR‐429, miR‐200c or miR‐141 mimics regulates protein expression of EMT markers. OVCAR‐3/TP GFP cells were transfected with negative control (nc), miR‐200a, miR‐200b, miR‐200c, miR‐429, or miR‐141 mimics. The EMT markers E‐cadherin (A), Vimentin (B), and Fibronectin (C) proteins were measured 24 h later by flow cytometry. The representative FACS histograms of 10,000 events per condition are shown in each panel. For easier interpretation of FACS histograms the results are presented as bar graphs (right panel) where y‐ax presents the fluorescence intensity. [file MOL2-9-1678-s002.jpg]

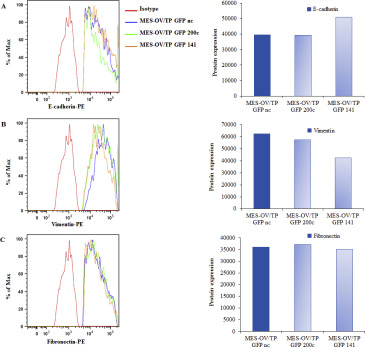

Supplement: Supplementary file 3 — Supplemental Figure 3 Transient transfection of MES‐OV/TP GFP cells with miR‐200c or miR‐141 mimics regulates EMT markers. MES‐OV/TP GFP cells were transiently transfected with miR‐200c or miR‐141 mimics. Cells were collected 48 h after the transfection, and expression of E‐cadherin (A), Vimentin (B), and Fibronectin (C) proteins was measured by flow cytometry. The representative FACS histograms of 10,000 events per condition are shown in each panel. For easier interpretation of FACS histograms the results are presented as bar graphs (right panel) where y‐ax presents the fluorescence intensity. [file MOL2-9-1678-s003.jpg]
